# Advances in mesenchymal stromal cell therapy for acute lung injury/acute respiratory distress syndrome

**DOI:** 10.3389/fcell.2022.951764

**Published:** 2022-08-10

**Authors:** Chang Liu, Kun Xiao, Lixin Xie

**Affiliations:** ^1^ School of Medicine, Nankai University, Tianjin, China; ^2^ Center of Pulmonary and Critical Care Medicine, Chinese People’s Liberation Army (PLA) General Hospital, Beijing, China; ^3^ Medical School of Chinese People’s Liberation Army (PLA), Beijing, China

**Keywords:** MSCs, acute lung injury, acute respiratory distress syndrome, therapy, mesenchymal stromal cells

## Abstract

Acute lung injury (ALI)/acute respiratory distress syndrome (ARDS) develops rapidly and has high mortality. ALI/ARDS is mainly manifested as acute or progressive hypoxic respiratory failure. At present, there is no effective clinical intervention for the treatment of ALI/ARDS. Mesenchymal stromal cells (MSCs) show promise for ALI/ARDS treatment due to their biological characteristics, easy cultivation, low immunogenicity, and abundant sources. The therapeutic mechanisms of MSCs in diseases are related to their homing capability, multidirectional differentiation, anti-inflammatory effect, paracrine signaling, macrophage polarization, the polarization of the MSCs themselves, and MSCs-derived exosomes. In this review, we discuss the pathogenesis of ALI/ARDS along with the biological characteristics and mechanisms of MSCs in the treatment of ALI/ARDS.

## Introduction

### ALI and ARDS

Acute lung injury (ALI) and acute respiratory distress syndrome (ARDS) refers to the diffuse injury of pulmonary capillary endothelium and alveolar epithelium caused by infection, shock, blood transfusion and other factors (Matthay et al., 2019a). Patients with mild hypoxemia (PaO2/FiO2≤300mmHg) are considered to have ALI, while those with more severe hypoxemia (PaO2/FiO2≤200mmHg) are considered to have the ARDS (Bernard et al., 1994).

**FIGURE 1:**
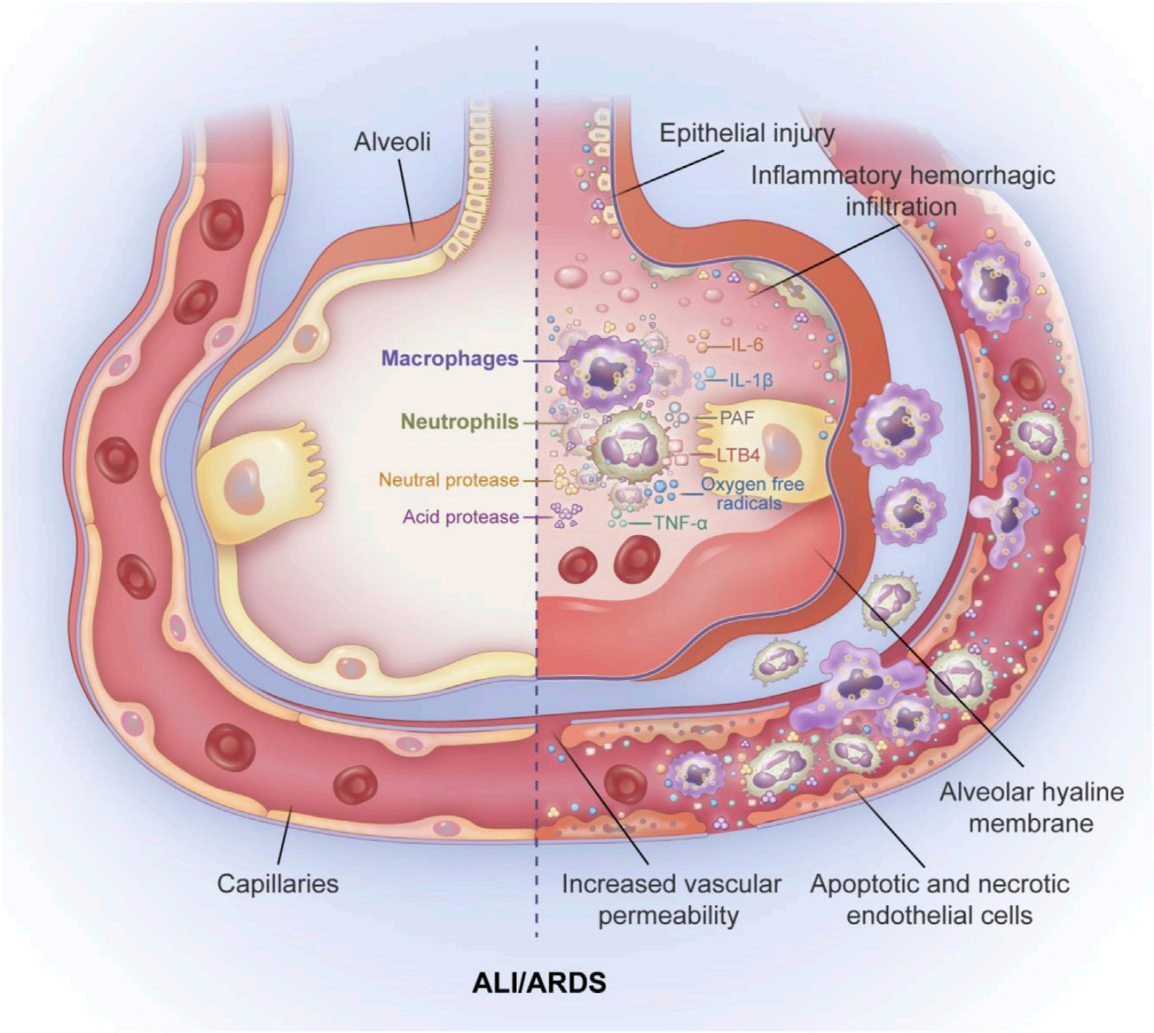
The pathological features of ALI/ARDS. The main pathological feature of ALI/ARDS is diffues injury of pulmonary capillary endothelial cells and pulmoary epithelial cells accompanied by alveolar hyaline membrane formation, increased pulmonary vascular permeability and inflamatory hemorrhagic infiltrate of alveolar space. The mechanism of increased alveolar capillary permeability is mainly the release of inflammatory factors and the aggregation of inflammatory cells. neutrophils and macrophages accumulate in the alveolar space and release large amounts of inflammatory cytokines TNF-α, IL-6, IL-1β. In addition, neutrophils can also release large amounts of oxygen free radicals, leukotriene B4 (LTB4), platelet activating factor (PAF), and various neutral and acidic proteases, leading to apoptosis and necrosis of alveolar epithelial cells and alveolar capillary endothelial cells, and ultimately to increased permeability of alveolar capillary endothelial cells.

ALI and its more severe form, ARDS, are critical illnesses that pose a threat to life and have high morbidity and mortality in critically ill patients (Rubenfeld et al., 2005). ALI/ARDS presents with acute or progressive hypoxic respiratory failure with severe inflammation of the respiratory tract (Ware and Matthay, 2000). The main pathophysiological characteristics of ALI/ARDS are imbalanced ventilation/blood flow ratio, decreased pulmonary compliance, and an increase in intrapulmonary shunts (Sweeney and McAuley, 2016). The main pathological feature is the diffuse injury (apoptosis) of pulmonary capillary endothelial cells and pulmonary epithelial cells (Figure 1) (Matthay and Zemans, 2011). In an international, multicenter, prospective cohort study, the prevalence of ARDS in patients admitted to the intensive care unit (ICU) was 10.4%, and the mortality rate for those with severe ARDS was 46.1% (Bellani et al., 2016). The current treatment for ALI/ARDS includes drug therapy and respiratory support therapy. Moreover, the use of a conservative fluid management strategy is suggested for all patients (Griffiths et al., 2019). Glucocorticoids have well-established anti-inflammatory and immunosuppressive effects; however, the potential benefits associated with corticosteroid therapy in patients with ARDS are not fully understood. A randomized controlled trial in Spain showed that the early use of dexamethasone in patients with moderate to severe ARDS was associated with significantly lower mortality and shorter duration of mechanical ventilation (Villar et al., 2020). Another multicenter randomized clinical trial revealed that patients with ARDS in the corticosteroid group had a higher number of ventilate-free days than the corticosteroid free group; however, no significant differences were observed in all-cause mortality at 28 days, ICU-free days during the first 28 days, and mechanical ventilation duration at 28 days (Tomazini et al., 2020). A retrospective study showed that early high-dose corticosteroid treatment had no effect on hospital mortality; moreover, high doses of steroids usually increase the risk of infection (Hu et al., 2021). A meta-analysis conducted by Lin et al. suggested that adjunctive treatment with glucocorticoids may reduce mortality in patients with ARDS, although further research is needed to determine the optimal dose and duration of steroid therapy (Lin et al., 2021). Mechanical ventilation therapies include pulmonary-protective mechanical ventilation, prone position ventilation, and extracorporeal membrane pulmonary oxygenation (Fan et al., 2018; Hadaya and Benharash, 2020). Despite the progress in treatments for ALI/ARDS, the mortality of ALI/ARDS patients remains high, and innovative treatment strategies are urgently needed.

### MSCs

MSCs, which are self-replicating and exhibit multi-directional differentiation, play a crucial role in tissue repair and can be used in the treatment of numerous diseases (Fu et al., 2019). MSCs are easy to obtain and exist in bone marrow, muscle, umbilical cord, adipose tissue, endometrial polyps, dental tissue, synovial fluid, skin, foreskin, Wharton’s jelly, placenta, dental pulp, gingiva, amnion, and menstrual blood (Klingemann et al., 2008). In addition, MSCs are found in nearly every vascularized tissue including the upper and lower respiratory tract (Hennrick et al., 2007). Lung-resident MSCs can interact with epithelial cells and promote alveolar cell growth, differentiation, and self-renewal (Lee et al., 2017). A study showed that lung-resident MSCs were smaller, had a higher proliferation rate, possessed a higher colony-forming capacity and had a different cytokine profile compared to bone marrow-MSCs (Rolandsson Enes et al., 2016).

According to the the minimal defining criteria of human MSCs established by the the Mesenchymal and Tissue Stem Cell Committee of the International Society for Cellular Therapy (ISCT) (Dominici et al., 2006): (Matthay et al., 2019a). MSCs are plastic adherent fibroblasts with osteogenic, chondrogenic and adipogenic differentiation capabilities. (Bernard et al., 1994). In addition, MSCs express the cell surface markers CD73, CD90 and CD105 and do not express hematopoietic and endothelial antigens (CD14 or CD11b, CD19 or CD79α, CD45, HLA-DR), and MSCs must be plastic-adherent under standard culture conditions. (Rubenfeld et al., 2005). MSCs must differentiate to osteoblasts, adipocytes and chondroblasts *in vitro*.

The characteristics of mesenchymal stromal cells from different sources are different (Table 1). Umbilical cord (UC)-MSCs displayed the highest proliferation rate with three to four times higher than that of adipose tissue (AD) and bone marrow (BM)-MSCs (Kim et al., 2018a). UC-MSCs have a faster osteogenesis compared to BM-MSCs under the same osteogenic conditions (Kern et al., 2006; Maqsood et al., 2020). The adipogenic differentiation ability of BM-MSCs and AD-MSCs was significantly higher than that of UC-MSCs (Kern et al., 2006; Maqsood et al., 2020). It has been described that AD-MSCs,BM-MSCs and UC-MSCs commonly expressed specific surface markers, but other antigens were differently expressed (Dominici et al., 2006). AD-MSCs typically expressed CD34 differentiation cluster, while UC-MSCs and BM-MSCs showed the negative expression for CD34 (Gonzalez-Vilchis et al., 2022). UC-MSCs expressed adhesion molecule markers (CD54, CD13, CD29, CD44) and they also expressed Tra-1-60, Tra-1-81, stage specific embryonic antigen-1 (SSEA-1) and alkaline phosphate. However, UC-MSCs did not express the immune response-related antigens involved in T lymphocyte activation, such as CD80, CD86 and CD40, CD40L and major histocompatibility complex (MHC) class II antigen HLA-DR (Thaweesapphithak et al., 2019; Stefańska et al., 2020). There may be different effects when using MSCs from different sources. In a hyperoxia-induced lung injury model, investigators found that Amniotic fluid MSCs had better therapeutic effects compared with UC-MSCs in relieving the pulmonary alveoli histological changes and promoting neovascularization (Xie et al., 2021). In this review, the sources of MSCs in the included literature were predominantly bone marrow, umbilical cord and adipose tissue.

**TABLE 1:** Characteristics of MSCs from different sources.

MSCs sources	Differentiation	Markers	Proliferation	References
Bone marrow	Adipocytes	The cell surface markers CD73, CD90 and CD105; BM-MSCs showed the negative expression for CD34 (Hematopoietic and endothelial antigens)	BM-MSCs have low proliferative capacity	(Dominici et al., 2006; Kern et al., 2006; Maqsood et al., 2020; Gonzalez-Vilchis et al., 2022)
	Osteoblasts chondrocytes			
	Cardiomyocytes			
	Hepatocytes			
	Muscles			
	Neuron			
	Embryonic tissues			
Adipose tissue	Adipocytes The adipogenic differentiation ability of BM-MSCs was significantly higher than that of UC-MSCs	The cell surface markers CD73, CD90 and CD105; AD-MSCs typically expressed CD34 differentiation cluster (Hematopoietic and endothelial antigens)	AD-MSCs are much more proliferative than BM-MSCs	Dominici et al. (2006); Kern et al. (2006); Maqsood et al. (2020); Gonzalez-Vilchis et al. (2022)
	Osteocytes			
	Chondrocytes			
	Muscle cells			
	The adipogenic differentiation ability of AD-MSCs was significantly higher than that of UC-MSCs			
Umbilical cord	Osteocytes, chondrocytes UC-MSCs have a faster osteogenesis compared to BM-MSCs under the same osteogenic conditions	The cell surface markers CD73, CD90 and CD105; UC-MSCs showed the negative expression for CD34 (Hematopoietic and endothelial antigens); UC-MSCs also expressed adhesion molecule markers (CD54, CD13, CD29, CD44) and Tra-1-60, Tra-1-81, stage specific embryonic antigen-1(SSEA-1) and alkaline phosphate. No expression of immune response-related antigens involved in T lymphocyte activation, such as CD80, CD86 and CD40, CD40L and major histocompatibility complex (MHC) class II antigen HLA-DR.	UC-MSCs displayed a stronger proliferation rate than AD-MSCs and BM-MSCs	Dominici et al. (2006); Kern et al. (2006); Thaweesapphithak et al. (2019); Maqsood et al. (2020); Stefańska et al. (2020); Gonzalez-Vilchis et al. (2022)

MSCs also exhibit an adherent growth pattern, and are relatively stable and easy to grow and culture (Pittenger et al., 1999). Due to these properties, MSCs are widely used in tissue repair. MSCs can secrete various growth factor to promote the regeneration of type II alveolar epithelial cells (Shi et al., 2021a). MSCs can also inhibit cytokine storm and decrease the number of infiltrated immune cells (Shi et al., 2021a). Therefore, MSCs can promote the repair of ALI/ARDS and have important application prospects in regenerative medicine (Laffey and Matthay, 2017).

MSCs can inhibit the proliferation of activated T cells (Di Nicola et al., 2002; Lee et al., 2020). MSCs can also regulate the proliferation and differentiation of B cells (Luz-Crawford et al., 2016), reduce the activity of natural killer cells (Bruno et al., 2016) and induce immune tolerance in dendritic cells (Lu et al., 2019) to ameliorate tissue inflammation. MSCs secrete a variety of cytokines that activate anti-apoptotic and pro-survival pathways, leading to tissue repair and regeneration (Harrell et al., 2019a; Eleuteri and Fierabracci, 2019). MSCs have been shown to reduce tissue inflammation and improve survival (Yu et al., 2019; Ishiuchi et al., 2020; Dos Santos et al., 2022). However, the mechanism by which MSCs repair ALI/ARDS is not fully understood.

## Biological roles of MSCs

The immune regulation mechanism of MSCs is complex and involves paracrine signaling (Lu et al., 2019), exosomes, cytokines (Zahari et al., 2017; Ha et al., 2020), homing and multi-directional differentiation (Harrell et al., 2019b). MSCs can regulate inflammation by influencing the phenotypes of macrophages and regulating the phenotypes of the MSCs themselves (Betancourt, 2013; Yuan et al., 2020). At present, research on MSCs involves a wide range of diseases: autoimmune diseases such as systemic lupus erythematosus (Wang et al., 2017a) and graft-versus-host disease (Zhao et al., 2019), cardiovascular disease such as atherosclerosis (Xing et al., 2020) and miocardial infarction (Deng et al., 2019), acute/chronic kidney disease (Yun and Lee, 2019), digestive system diseases such as liver diseases (Wang et al., 2019a) and inflammatory bowel disease (Yang et al., 2019), respiratory disease such as chronic obstructive pulmonary disease (COPD) (Ridzuan et al., 2021), asthma (Dong et al., 2021) and pulmonary hypertension (Klinger et al., 2020). MSCs have numerous medical applications and are the most widely used stem cells in the field of regenerative medicine.

### MSCs homing

**FIGURE 2:**
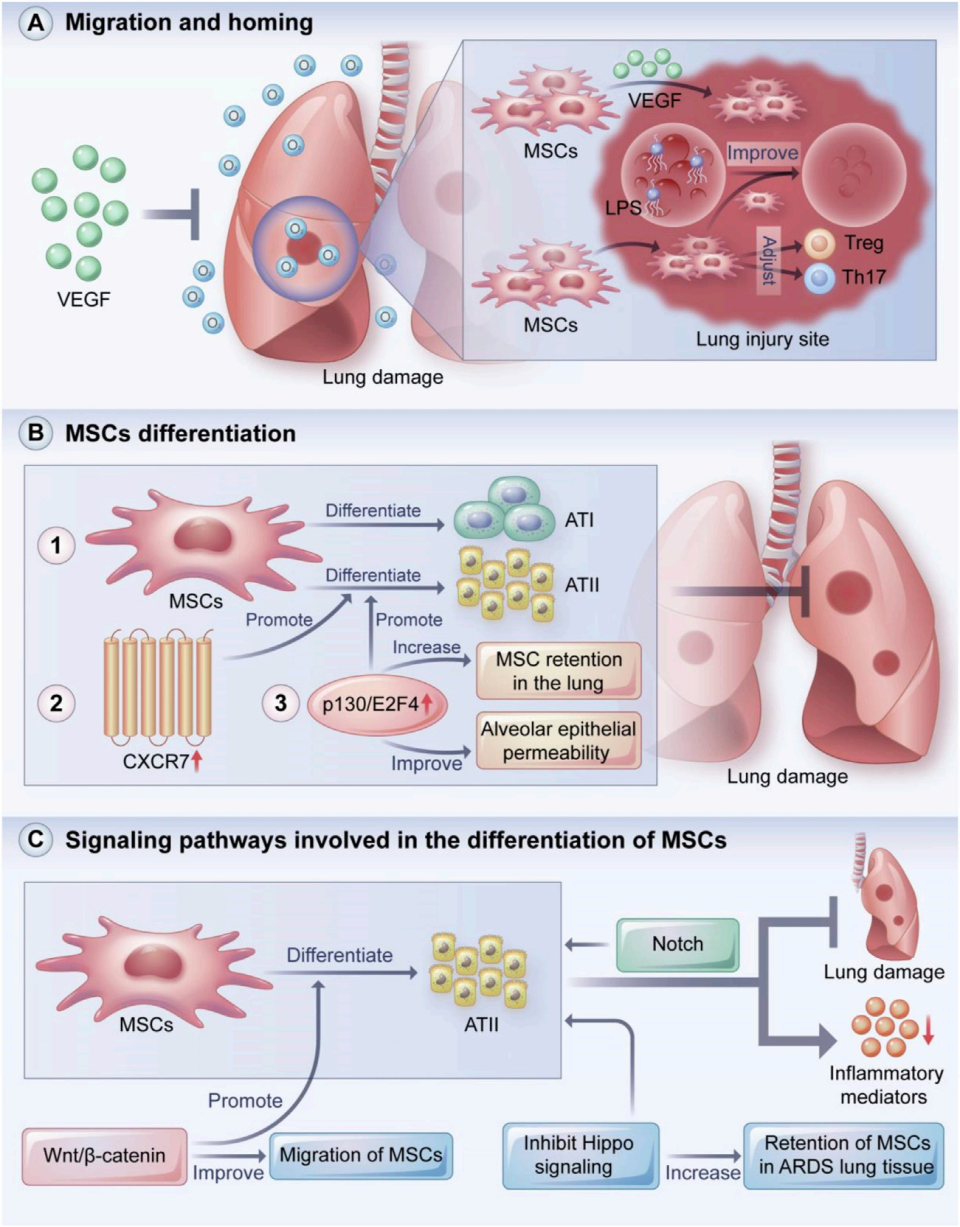
MSCs migration and homing, MSCs differentiation and signaling pathways involved in the differentiation of MSCs. VEGF plays a key role in recruiting MSCs to damaged tissues, thereby preventing hyperoxia-induced lung injury. In addition, MSCs can migrate and remain at the injury site, regulate the balance of Treg and Th17 cells, and ameliorate LPS-induced lung injury. MSCs can protect the lung from injury by localizing to damaged lung tissue and directionally differentiating into ATI and ATII cells. Overexpression of CXCR7 promotes differentiation of MSCs to ATII cells, and overexpression of p130/E2F4 enhances differentiation of MSCs to ATII cells and increases retention of MSCs in the lung and improves alveolar epithelial permeability. Wnt/β-catenin, Hippo and Notch signaling pathways play important roles in the differentiation, migration and tissue retention of MSCs. VEGF: vascular endothelia growth factor; Treg: regulatory T cells; ATI: type I alveolar epithelial cells; ATII: type II alveolar epithelial cells. **(A)** MSCs migration and homing, **(B)** MSCs differentiation and **(C)** signaling pathways involved in the differentiation of MSCs.

MSCs can migrate to damaged tissues and play a protective role in lung injury through homing and differentiation (Figure 2A) (Harrell et al., 2019b). MSC homing can be defined as the process of capturing MSCs in the vascular system of the target tissue followed by the migration across the vascular endothelial cells to the target tissue (Karp and Leng Teo, 2009).

The stem cell nest is a special microenvironment that forms the foundation of stem cells. The homing process of stem cells is associated with a series of ligands and their receptors, including various chemokines, adhesion molecules, and growth factors (Ringe et al., 2007; Zhou et al., 2017), such as CXC chemokine receptors (CXCRs), monocytes chemotactic proteins (MCPs), adhesion molecules, stromal cell-derived factor one (SDF-1), vascular endothelial growth factor (VEGF), and hepatocyte growth factor (HGF).

SDF-1, which is also known as CXCL12, is a CXC subfamily of the chemokine family. CXCR4, the most studied SDF-1 receptor, was first identified as a cytokine secreted by mouse bone marrow MSCs (BMSCs) and is expressed in variety of cells (Moll and Ransohoff, 2010; Pozzobon et al., 2016; Mousavi, 2020). The SDF-1/CXCR4 axis modulates MSC migration, chemotaxis, and homing to injury sites; thus, CXCR4 is a key signal that is highly conserved in evolution and mediates the migration and distribution of stem cells (Chen et al., 2020a). SDF-1 is the strongest predictor of MSCs migration and homing in myocardial biopsies from patients with viral-negative inflammatory cardiomyopathy (Schmidt-Lucke et al., 2015). The upregulation of CXCR4 can enhance the migration and homing capability of MSCs (Shi et al., 2007a; Pozzobon et al., 2016). The targeted expression of SDF-1α after myocardial infarction increased the engraftment of BMSCs into infracted myocardium (Ghadge et al., 2011). This effect is beneficial for cardiomyocyte survival, neovascularization, and cardiac function. Thus, the SDF-1/CXCR4 axis appears to be a promising new therapeutic target.

MCP-1 (CCL2) and MCP-3 (CCL7) are members of the chemokine family that are chemotactic for a variety of cells and exert regulatory effects by binding to specific receptors. Guo et al. reported that MCP-1 promoted the myocardial homing of MSCs in dilated cardiomyopathy (DCM), while MSCs transplantation reduced myocardial fibrosis in DCM mice and improved cardiac function (Guo et al., 2013). Scherk et al. reported that the local overexpression of MCP-3 recruited MSCs to damaged tissue sites and improved cardiac remodeling independent of cardiomyocyte regeneration (Schenk et al., 2007).

Although MSCs are relatively easy to obtain and propagate via culturing, their implantation into target tissues during autologous transplantation remains a major challenge. MSCs and the extracellular matrix bind to cell adhesion molecules via the expression of cell adhesion molecule ligands, which mediate the homing of stem cells to targets. Adhesion molecules with important functions associated with the adhesion of MSCs to endothelial tissues include P-selectin and integrins (Brooke et al., 2008; Kot et al., 2019). Kumar et al. demonstrated that the transient and ectopic expression of alpha4 integrin on MSCs significantly increased bone homing in an immunocompetent mouse model (Kumar and Ponnazhagan, 2007). This may broadly benefit in targeted therapies for osteopenic bone defects and cancer bone metastasis. Liao et al. reported that P-selectin glycoprotein ligand-1 and sialyl-Lewis X greatly enhanced the homing ability of MSCs to the inflammation-affected spinal cord via mRNA transfer, thereby improving the therapeutic effect of experimental autoimmune encephalomyelitis in mice (Liao et al., 2016). In a rat model of pulmonary arterial hypertension, platelets and their P-selectin promoted the adhesion of MSCs to extracellular matrix collagen, demonstrating the homing of MSCs to the pulmonary vasculature (Jiang et al., 2012). The authors further demonstrated that cell homing was abolished by an anti-P-selectin antibody and the GPIIb/IIIa inhibitor tirofiban (Liao et al., 2016).

Ahn et al. reported that VEGF secreted by MSCs prevented hyperoxic lung injury (Ahn et al., 2018). The VEGF secreted by the MSCs was involved in the formation of microtubules and the development of angiogenesis by binding to the corresponding receptor and activating signaling cascades; the VEGF also played a crucial role in the recruitment of stem cells to damaged tissues. HGF has a variety of biological effects, including the inhibition of apoptosis and promotion of cell proliferation (Nakamura and Mizuno, 2010). Shams et al. found that MSCs pretreated with HGF significantly improved MSC homing in the liver compared to untreated MSCs (Shams et al., 2015). The glycogen storage was restored, and the levels of collagen, alkaline phosphatase, and bilirubin were significantly reduced. In a rat model of acute liver failure (ALF), the overexpression of c-Met promoted MSC homing to damaged liver sites, and this effect was dependent on HGF (Wang et al., 2017b). Results showed that the liver function and survival rates were elevated in the ALF rats. Sun et al. reported that the stable expression of HGF in BMSCs facilitated MSC homing and further improved their therapeutic effect in liver fibrosis (Sun et al., 2020). Wang et al. demonstrated that MSCs in the lungs can migrate and be retained at injured sites, regulate the balance of regulatory T cells (Tregs) and Th17 cells, and ameliorate lipopolysaccharide (LPS)-induced lung injury (Wang et al., 2019b).

### Multi-directional differentiation

MSCs can differentiate into alveolar epithelial or airway epithelium cells under different *in vitro* induction conditions (Figure 2B). For instance, Spees et al. co-cultured human MSCs with heat shock-treated small airway epithelial cells and found that a fraction of the MSCs rapidly differentiated into epithelioid cells and restored the epithelial structure (Spees et al., 2003). Rojas et al. reported that BMSCs protected the lung from injury by localizing in damaged lung tissue and manifesting a lung cell phenotype (Rojas et al., 2005). Shao et al. reported that the overexpression of CXCR7 promoted MSC differentiation toward type II alveolar epithelial (ATII) cells to ameliorate lung injury (Shao et al., 2019). In an LPS-induced ARDS mouse model, the overexpression of p130/E2F4 increased MSC retention in the lung, increased MSC differentiation into ATII cells, and improved alveolar epithelial permeability (Zhang et al., 2019).

BMSCs can limit bleomycin-induced pulmonary inflammation and collagen deposition by differentiating into ATII cells, which are thought to function as stem cells in the lung (Ortiz et al., 2003). Some signaling pathways are also involved in MSC differentiation (Figure 2C). Cai et al. found that the Wnt/β-catenin pathway promoted the differentiation of MSCs into ATII cells and improved MSC migration, suggesting that the Wnt/β-catenin pathway is a key mechanism in the treatment of mice with ALI/ARDS (Cai et al., 2015). Li et al. found that inhibiting Hippo signaling increased the retention of MSCs in ARDS lung tissue and the differentiation of MSCs into ATII cells (Li et al., 2019). In ATII cells induced by human umbilical cord MSCs, Liu et al. found that β-catenin signaling was involved in the protective effect against apoptosis in mice with pulmonary fibrosis (Liu et al., 2021a). Liang et al. found that BMSCs significantly reduced the production of inflammatory mediators and attenuated lung injury by differentiating into ATII cells, which may be associated with the Notch signaling pathway (Liang et al., 2019). Lastly, a study demonstrated an important relationship between the degree of lung injury and the phenotypic change from BMSCs to lung epithelial cells (Herzog et al., 2006). That is, transplanted BMSCs adopted an epithelial phenotype in the lung only in the presence of significant lung damage.

### Anti-inflammatory effect

**FIGURE 3:**
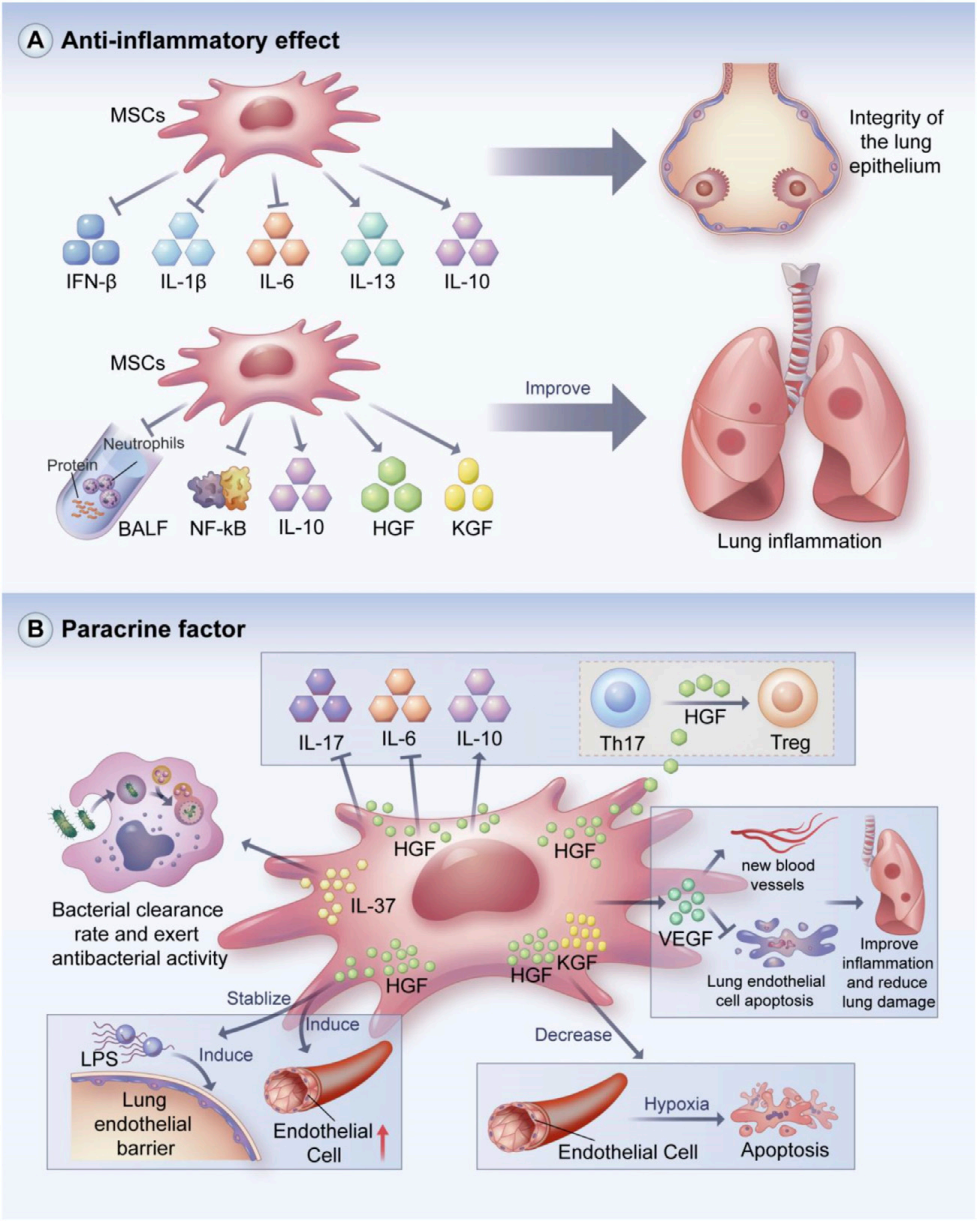
Anti-inflammatory and paracrine effects of MSCs. MSCs can maintain the integrity of pulmonary epithelium by reducing pro-inflammatory factors and increasing the secretion of anti-inflammatory factors. In addition, MSCs can reduce protein levels and neutorphil recruitment in BALF, inhibit NF-kB-related molecular activity, and enhance the secretion of HGF and KGF to improve lung inflammation. MSCs can exert various regulatory effects through paracrine soluble cytokines, such as anti-bacterial avtivity, promotion of angiogenesis, inhibition of lung endothelial cell apoptosis, maintenance of lung endothelial barrier stability and immunomodulatory effects. BALF: bronchoalveolar lavage fluid; HGF: hepatocype growth factor; KGF: Keratinocyto growth factor. **(A)** Anti-inflammatory and **(B)** paracrine effects of MSCs.

MSCs have powerful anti-inflammatory effects and can promote the repair of lung tissue injury (Figure 3A). Loy et al. demonstrated the anti-inflammatory effects of MSCs and found that MSCs could maintain the integrity of the lung epithelium by reducing the expressions of pro-inflammatory factors such as IL-6, IFN- β, and IL-1β and enhancing the expressions of anti-inflammatory factors such as IL-10 and IL-13. (Loy et al., 2019). MSCs secrete IL-1 receptor antagonist (IL-1Ra), which can reduce the activation of inflammasomes and inhibit IL-1β production in macrophages to alleviate ALI (Harrell et al., 2020).

BMSCs can attenuate lung injury in ALI mice by reducing protein levels and neutrophil recruitment in bronchoalveolar lavage fluid (BALF) and improving pulmonary histological changes; BMSCs can also decrease the protein levels of pro-inflammatory cytokines including IL-1β, IL-6, and TNF-α (Huh et al., 2018). This study also showed that the concentration of macrophage inflammatory protein did not decrease with the MSCs injection (Huh et al., 2018). Moreover, MSCs can reduce NF-kB nuclear transfer and enhance the secretion of HGF, IL-10, and keratinocyte growth factor (KGF) to reduce lung inflammation (Chen et al., 2019a).

Signaling pathways play a key role in the anti-inflammatory process. BMSCs reduced LPS-induced increases in inflammatory cell counts and promoted the expressions of TNF-α, IL-1β, and IL-6 in BALF (Lei et al., 2021). The enhanced expression of Nrf2 and HO-1 and decreased expression of NOD-like receptor protein 3 (NLRP3) and caspase-1 was observed in lung tissue. Therefore, BMSCs may play an anti-inflammatory effect partly through the Nrf2/HO-1-dependent NLRP3 pathway (Lei et al., 2021). MSC- conditioned medium can ameliorate LPS-induced ALI, potentially by suppressing inflammation and oxidative stress through the Nrf2/NF-kB signaling pathway (Tang et al., 2021a). This may be related to a variety of cytokines and growth factors contained in conditioned medium. Moreover, MSCs can home to sites of lung injury and reduce epithelial permeability probably by blocking TGF-β1 and wnt3/β-catenin signaling (Zhang et al., 2016). Our group demonstrated that MSCs can attenuate lung injury and reverse fibrosis by suppressing the NF-kB and hedgehog pathways (Xiao et al., 2020).

### Action of paracrine factors

MSCs can perform immunomodulation by secreting soluble factors such as cytokines and growth factors, which play important roles in the stabilization of the immune internal environment (Figure 3B) (Gebler et al., 2012; Harrell et al., 2019c; Alvites et al., 2022). For example, IL-37 secreted by MSCs can enhance bacterial clearance and exert antibacterial activity (Krasnodembskaya et al., 2010). MSCs can suppress harmful immune responses and promote tissue repair and regeneration by increasing IL-10 expression (Lindner et al., 2021). Arg-1 released from MSCs can restore protein permeability in alveolar epithelial cells (Fang et al., 2010).

MSCs can regulate the immune response in animal models of ALI/ARDS through paracrine action. For example, Yang et al. found that MSC-derived VEGF can ameliorate inflammatory responses and reduce lung injury by promoting neovascularization and protecting pulmonary endothelial cells from apoptosis (Yang et al., 2016). Umbilical cord MSCs suppressed macrophage inflammatory responses in ALI mice, possibly through the paracrine secretion of PGE2 (Zhu et al., 2017). Moreover, MSCs have a stabilizing effect on LPS-induced pulmonary endothelial barrier dysfunction through the paracrine secretion of HGF (Hu et al., 2016; Shi et al., 2021b; Geng et al., 2021). The main mechanisms by which HGF restores the integrity of the endothelial cell monolayer are the remodeling of endothelial intercellular connections, decreasing the expression of caveolin-1 protein, and inducing human pulmonary microvascular endothelial cell proliferation (Chen et al., 2015).

Li et al. found that BMSCs exhibited anti-fibrotic effects in silica-induced pulmonary fibrosis, which may be related to paracrine mechanisms rather than differentiation, as indicated by the elevated levels of HGF and KGF were in the BMSC group compared with silica-treated group (Li et al., 2017). Verghese et al. found that HGF and KGF were active in the alveolar space in the early stage of ALI, which may mediate the early events of lung repair (Verghese et al., 1998). The authors also found an increased level of HGF in edema fluid, which might have prognostic value in the early stage of ALI. Bernard et al. confirmed that MSCs reduced hypoxia-induced apoptosis in alveolar epithelial cells via the paracrine secretion of HGF and KGF, which decreased the accumulation of reactive oxygen species (ROS) and hypoxia-induced factor 1α (Bernard et al., 2018).

MSCs can alleviate lung inflammatory damage by modulating TLR4 signaling, which may be partly associated with the increased secretion of KGF and Ang-1 by MSCs under inflammatory conditions (Chen et al., 2019b). Chen et al. reported that MSCs significantly suppressed the expressions of IL-17 and IL-6, increased IL-10 expression, and induced the conversion of differentiated Th17 cells into functional Treg cells, which was ascribed to the secretion of HGF by MSCs (Chen et al., 2020b). These results suggest that HGF secreted by MSCs has immunomodulatory effects. Dong et al. found that MSC infusion may limit fibroblast activation to exert an anti-fibrotic effect, which was partly attributed to the secretion of endogenous HGF and PGE2 accompanied by decreases in TGF-β1 and TNF-α expression; this effect is known as the paracrine effect of MSCs in tissue repair (Dong et al., 2015). Nakajima et al. revealed that MSCs treatment decreased the levels of IL-18, TNF-γ, and TNF-α, increased the levels of IL-4 and HGF, and reduced the wet-to-dry weight ratio of lung tissue (Nakajima et al., 2019). These results suggest that HGF is a key paracrine factor released by MSCs. Wang et al. reported that MSCs treatment decreased endothelial permeability and IL-6 production and increased IL-10 production (Wang et al., 2017c). However, these effects of MSCs treatment were inhibited by HGF knockout, demonstrating that HGF may play a crucial role in the function of MSCs.

### Macrophage phenotype regulation

**FIGURE 4:**
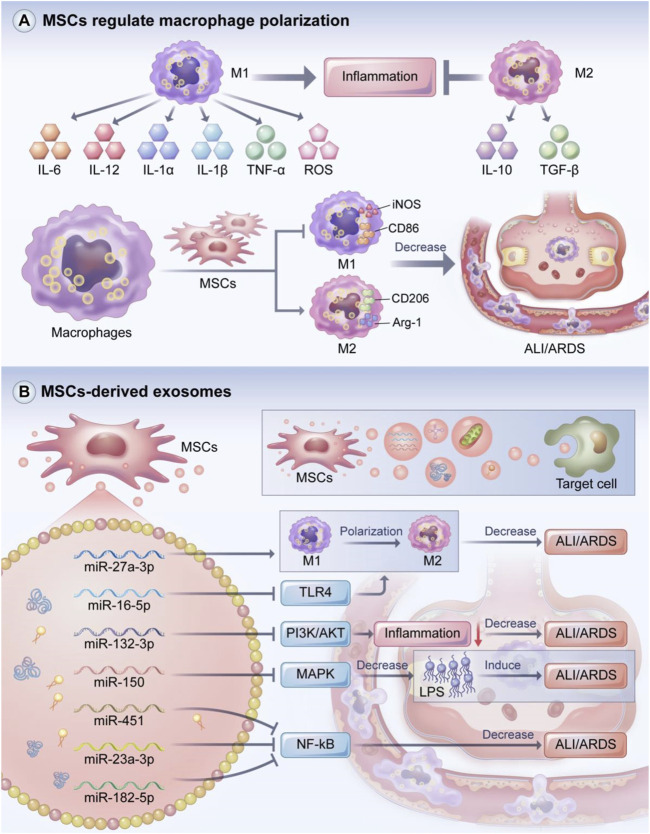
MSCs regulate macrophage polarization and the role of MSCs-derived exosomes. Macrophages include M1 and M2 macrophage phenotypes, with M1 macrophages exerting a pro-inflammatory effect and M2 macrophages acting as an inhibitor of inflammation. MSCs can promote M2 macrophage polarization while suppressing M1 macrophage polarization to attenuate the inflammatory response in ALI/ARDS. MSCs-derived exosomes can transfer mRNA, miRNA, proteins, lipids and even mitochondria to target cells and tissues, playing a key role in immune regulation of inflammatory responses. The miRNA carried in MSCs-derived exosomes can promote M2 macrophage polarization to alleviate ALI/ARDS. In addition, MSCs-derived exosomal miRNA can improve ALI/ARDS by regulating PI3K/AKT, MAPK, NF-kB and other signaling pathways. **(A)** MSCs regulate macrophage polarization and the role of **(B)** MSCs-derived exosomes.

Macrophages, which are a heterogeneous population of immune cells, can be divided into two main subpopulations based on their biomarkers and functions (Figure 4A) (Johnston et al., 2012). M1 macrophages produce pro-inflammatory responses and secrete pro-inflammatory factors such as IL-6, IL-12,IL-1α, IL-1β, TNF-α, and ROS. However, the excessive release of pro-inflammatory cytokines and the recruitment of inflammatory factors are detrimental to the lungs and contribute to ALI/ARDS. M2 macrophages are immunoregulatory cells that secrete IL-10 and TGF-β. M2 macrophages can also suppress the level of pro-inflammatory cytokines, which may promote host tissue repair, reduce alveolar epithelial cell damage, and increase lung barrier function after inflammation (Herold et al., 2011; Murray et al., 2014; Patel et al., 2017; Huang et al., 2018).

M1 macrophages are usually induced by Th1 cytokines such as LPS and IFN-γ (Sica et al., 2015; Bashir et al., 2016). Song et al. found that excessive neutrophil extracellular traps (NETS) led to severe inflammation and increased the levels of M1 markers including IL-6, IL-1β, TNF-α, and Myeloperoxidase (MPO) in BALF, suggesting that NETS can promote ARDS inflammation in the acute phase (Song et al., 2019). M2 macrophages are usually induced by Th2 cytokines. For instance, IL-4 and IL-13 may promote M2 macrophage polarization partly through JAK1/STAT6 pathway, thereby reducing inflammation (He et al., 2020), and IL-10 may regulate M2 polarization by activating STAT3 through the IL-10 receptor (Gordon, 2003; Porta et al., 2015). Other cytokines such as IL-33 can also induce polarization to the M2 phenotype (You et al., 2020).

MSCs can regulate macrophage phenotype (Figure 4A). In Japanese encephalitis, MSCs reduced the inflammatory response and improved survival by inhibiting glial cell activation, promoting the transformation of M1 macrophages to M2 macrophages, and decreasing neuronal cell death (Bian et al., 2017). Huang et al. found that pancreatic tissue macrophages exhibited significantly increased expressions of CD163 and Arg one and significantly decreased expressions of CD86 and iNOS; based on these results, they hypothesized that MSCs regulate macrophage polarization and reduce pancreatic injury by secreting TSG-6 (Huang et al., 2021). Our group found that MSCs induced macrophage polarization towards the anti-inflammatory M2 phenotype and reduced insulin resistance in rates with type 2 diabetes induced by the pro-inflammatory M1 phenotype (Xie et al., 2016).

MSCs also play a key role in ALI/ADRS by regulating macrophage phenotype polarization. Deng et al. demonstrated that MSCs can treat LPS-induced ARDS by inhibiting M1 polarization, promoting M2 polarization, and suppressing the expression of hypoxia transcription factor-1, which is important in controlling innate immune function and regulating macrophage polarization (Nizet and Johnson, 2009; Deng et al., 2020). Chen et al. found that when co-cultured with MSCs, macrophages transformed into an the anti-inflammatory M2 phenotype characterized by the M2 polarization markers Arg-1 and CD163 (Chen et al., 2018). Co-culturing with MSCs also suppressed the production of the pro-inflammatory cytokines MCP-1, IL-1, IL-6, and TNF-α and increased the expression of the anti-inflammatory cytokine IL-10. The mechanism of these effects might be related to the paracrine factors TGF-β3 and thrombospondin-1. Lv et al. found that heat shock-pretreated MSCs (HS-MSCs) modulated the balance between M1 and M2 macrophages to reduce the levels of inflammatory cytokines, increase the expression of the anti-inflammatory marker CD206, and inhibit NLRP3 inflammasome activation (Lv et al., 2021).

### MSCs polarization

The heterogeneity of MSCs has been demonstrated in terms of growth characteristics, cell surface marker expression, differentiation potential, and immune regulation (Phinney, 2012). Interestingly, the inflammatory microenvironment has a profound effect on MSC-mediated immunomodulation. For example, an inflammatory microenvironment can significantly alter the immunosuppressive potential of MSCs, mainly through changes in the expressions of adhesion molecules and inflammatory cytokines (Ren et al., 2008; Prasanna et al., 2010; Ren et al., 2010). MSCs are sensors of inflammation; thus, the presence of an inflammatory microenvironment and interactions with innate and adaptive immune system components can lead to MSC polarization into pro-inflammatory MSC1 and anti-inflammatory MSC2 cells, similar to macrophage polarization to the pro-inflammatory M1 phenotype or anti-inflammatory M2 phenotype (Waterman et al., 2010; Bernardo and Fibbe, 2013).

MSCs modulate the immune system in a manner that depends on their activation status (Uccelli et al., 2008). Cytokine concentration can influence the immunosuppressive function of MSCs (Li et al., 2012). When the organism is externally injured, the site of injury releases large amounts of inflammatory cytokines that induce macrophages to polarize into the M1 phenotype. The M1 macrophages then secrete large amounts of pro-inflammatory factors such as IFN-γ and TNF-α. IFN-γ is a robust stimulator that activates MSCs to exert their immunosuppressive functions as MSC2 (Wang et al., 2014). Together with TNF-α, IL-α, and IL-1β, IFN-γ can enhance the immunosuppressive capacity of MSC2 by promoting the expressions of numerous immunomodulatory factors such indoleamine 2,3-dioxygenase (IDO), NO, and PGE2, which in turn reduce lymphocyte function and mitigate tissue injury caused by excessive immune response (Ren et al., 2008; Krampera, 2011; Munn and Mellor, 2013; Szabó et al., 2015). MSCs cannot be activated into an immunosuppressive phenotype when the concentration of the inflammatory factor IFN-γ is low. MSCs are activated in the pro-inflammatory MSC1 form, which can secrete a variety of inflammatory cytokines that recruit inflammatory cells to the site of injury, which amplifies the inflammatory reaction and induces tissue damage (Waterman et al., 2010; Wobma et al., 2018). In some cases, the concentration of cytokines is sufficient to upregulate chemokine secretion, but not sufficient to induce significant expression of iNOS or IDO. In this situation, in the absence of the immunosuppressive effects of iNOS or IDO, chemokine-recruited lymphocytes can accumulate uninhibitedly near the MSCs, leading to more severe inflammation (Renner et al., 2009). MSCs inhibited T cell proliferation when conditions were rendered proinflammatory by adding concentrations of proinflammatory cytokines. (Renner et al., 2009). Application of anti-inflammatory cytokines like IL-10 abrogated the suppressive effect of MSCs (Renner et al., 2009). Therefore, MSCs play a role in balancing the immune inflammatory response.

Toll-like receptors (TLRs) are pattern recognition receptors that belong to type I transmembrane proteins containing conserved motifs known as leucine-rich repeats and toll/interlukin-1 receptor domains (Nie et al., 2018). TLR activation has an important correlation with MSCs polarization. To date, 10 TLR analogs have been identified in humans. Among them, TLR3 and TLR4 are consistently highly expressed in human MSCs (Raicevic et al., 2010). TLRs are expressed on the surfaces of endothelial cells, monocytes/macrophages, neutrophils, and dendritic cells; TLRs also play an important role in maintaining the homeostasis of the immune response (Takeda and Akira, 2005). Kim et al. demonstrated that polyinosinic-polycytidylic acid specifically aggregated with TLR3 on the surfaces of MSCs, promoted the polarization of MSCs toward MSC2, and induced the secretion of the inflammatory factor PGE2 (Kim et al., 2018b). TLR3 stimulation was found to enhance the inhibition of lymphocyte proliferation by BMSCs, which was associated with induction of IDO expression (Opitz et al., 2009; Chen et al., 2013; Munn and Mellor, 2013). MSC2 also induced the production and activation of Tregs and reduced the level of immune activation (Burr et al., 2013; Ma et al., 2014). Shi et al. reported that LPS promoted the activation of TLR4 on the surfaces of MSCs, which in turn promoted the polarization of MSCs toward MSC1 and induced the secretion of multiple inflammatory cytokines and chemokines (Shi et al., 2007b). TLR4 stimulation has been shown to decrease the immunosuppressive potential of MSCs, enhance the immune response characterized by T cells, and induce inflammatory response (Raicevic et al., 2010; Raicevic et al., 2011; Sangiorgi et al., 2016).

In summary, research on TLR expression in MSCs has revealed a connection between TLR signaling and MSC-mediated immunoregulatory functions. Pro-inflammatory signals can be transmitted to MSCs via TLR4, leading to differentiation into the MSC1 phenotype. In contrast, anti-inflammatory cytokines can be provided via TLR3, leading to differentiation into the MSC2 phenotype.

### MSC-derived exosomes

According to the latest definition of the International Society of Extracellular Vesicles (ISEV), the calssification of EVs are based on (Matthay et al., 2019a). physical characteristics: such as density (low, middle, high, with each range defined), or size [“small EVs” (<200 nm) and “medium/large EVs” (>200 nm)]; (Bernard et al., 1994). Biochemical composition: CD63^+^/CD81^+^-EVs, Annexin A5-stained EVs, etc. (Rubenfeld et al., 2005). Cell of origin: podocyte EVs, hypoxic EVs, large oncosomes, apoptotic bodies (Théry et al., 2018). In addition, MSC-EVs should be defined by quantifiable metrics to identify the cellular origin of the sEVs in a preparation, presence of lipid-membrane vesicles, and the degree of physical and biochemical integrity of the vesicles (Witwer et al., 2019).

Generally, EVs are classified into three categories based their biogenesis. Exosomes are generated from multivesicular bodies (MVBs) that are formed by internal budding of the endosomal membrane. MVBs can directly fuse with cellular plasma membrane and are released into the extracellular environment as exosomes (Raposo and Stoorvogel, 2013). The endosomal sorting complex required for transport (ESCRT) plays a crucial role in driving exosomal biogenesis (Juan and Fürthauer, 2018). Microvesicles are formed by external budding of the cell membrane in different cell types (Hanson and Cashikar, 2012). Apoptotic bodies are also large-size vesicles and they originate specifically from apoptotic cells (Caruso and Poon, 2018). Exosomes contain large amounts of annexins, tetraspanins such as CD63, CD81, and CD9, and heat-shock proteins, including Hsp60, Hsp70, and Hsp90. They also express Alix, tumor susceptibility gene 101 (Tsg101) (Raposo and Stoorvogel, 2013).

MSCs have short survival times at injury sites (Copland et al., 2009; Katsha et al., 2011; Li et al., 2016). Some studies have suggested that MSCs primarily exert their therapeutic function through paracrine secretion (Fu et al., 2019; Chang et al., 2021). However, MSCs have the possibility of tumorigenesis and poor differentiation (Caldas et al., 2017). Some studies indicated that sarcoma can evolve from MSCs cultures (Tolar et al., 2007; Xu et al., 2012). As the smallest subpopulation of extracellular vesicles, exosomes are membrane vesicles that are secreted by most cells, have a lipid bilayer membrane structure, and are important for mediating paracrine secretion (Mäger et al., 2013; Yáñez-Mó et al., 2015; Tang et al., 2021b). Exosomes contain miRNAs that regulate receptor cell functions. The miRNAs encapsulated in exosomes are strictly regulated by various pathophysiological stimuli and the microenvironment (Jelonek et al., 2016; Yu et al., 2016). MSC exosomes can transfer substances such as mRNAs, miRNAs, proteins, lipids, and even mitochondria to target cells and tissues, leading to changes in gene expression and target cell behavior; in this way, exosomes participate in intercellular communication and play a key role in the immune modulation of inflammatory response (Figure 4B) (Kourembanas, 2015; Tkach and Théry, 2016; Gong et al., 2017; Paliwal et al., 2018; Harrell et al., 2019d).

MSC-derived exosomes are critical in mitigating severe inflammatory response, especially in ALI/ARDS (Figure 4B). Thus, MSC-derived exosomes may be considered in the development of cell-free strategies for the treatment of ALI/ARDS. MSCs-derived exosomal miR-22-3p attenuated oxidative stress response and inhibited the activity of NF-kB signaling pathway-related molecules by reducing the expression of frizzled class receptor 6 (FZD6), thus attenuating ALI (Zheng et al., 2021). Wang et al. demonstrated that NF-kB1 was a target of miR-27a-3p, and the transfer of miR-27a-3p to alveolar macrophages promoted M2 macrophage polarization, thereby attenuating ALI (Wang et al., 2020). These results suggest that miR-27a-3p, which targets NF-kB1, is a key regulator of M2 polarization.

Liu et al.used a rat model of LPS-induced ALI, injected them with exosomes via the tail vein or trachea, and observed the degree of lung injury (Liu et al., 2021b). They found MSC-derived exosomal miR-384-5p attenuated lung injury by targeting Beclin-1 to alleviate impaired autophagy in alveolar macrophages. These results suggest that miR-384-5p is a promising therapeutic approach for ALI/ARDS due to its pulmonary protective effect. Tian et al. found that exosomal miR-16-5p promoted macrophage polarization and mitigated lung injury in mice models by inhibiting TLR4, while the overexpression of TLR4 impaired the therapeutic effect of miR-16-5p (Tian et al., 2021). In an LPS-stimulated ALI model, Liu et al. found that enhancing miR-132-3p expression reduced the inflammatory response and suppressed cell apoptosis via the targeting of TNF receptor-associated factor 6 and the inhibition of PI3K/AKT signaling by exosomal miR-132-3p (Liu et al., 2021c). Xu et al. found that LPS increased apoptosis and the expression of MAPK signaling-related protein, while exosomal miR-150 suppressed these increases (Xu et al., 2022). The authors also found that exosomal miR-150 attenuated lung inflammation, alleviated pulmonary edema, and maintained the integrity of the alveolar space, suggesting that exosomal miR-150 attenuates LPS-induced ALI via the MAPK pathway. The transfection of miR-150 antagomirs into MSCs partly reversed the immunomodulatory effect of MSC-derived exosomes (Xu et al., 2022). Liu et al. transferred miR-451 into burn-induced ALI rats and found that the expressions of IL-6,TNF-α, and IL-1β along with NF-kB signaling pathway proteins were decreased; these effects were reversed when miR-451 expression was suppressed (Liu et al., 2019). Chen et al. injected MSC-derived exosomes into the tail veins of ALI rats and found that the MSC-derived exosomes inhibited histopathological changes and improved pulmonary microvascular permeability (Chen et al., 2021). The therapeutic mechanism of these effects might be related to the inhibition of phosphorylation in the MAPK/NF-kB pathway and the degradation of IkB. Our group found that the delivery of miR-182-5p and miR-23a-3p via MSC-derived exosomes into LPS-treated MLE-12 cells improved LPS-stimulated ALI and inhibited the NF-kB and hedgehog signaling pathways by silencing Ikbkb and IKKβ, thereby ameliorating the progression of LPS-stimulated ALI and pulmonary fibrosis (Xiao et al., 2020).

A study reported that only a fraction of the miRNAs identified in MSCs were secreted in MSC exosomes (Albanese et al., 2021). EVs cargo such as miRNA probably did not participate in a biologically active transfer at physiologically relevant level (Chen et al., 2010). Whether exosome miRNA plays a role in mediating the therapeutic efficacy of MSC exosomes, it depends on the presence of a biologically relevant concentration of the miRNA. However, miRNAs are not present in sufficient quantities in a typical exosome dose to elicit a biologically relevant response (Chevillet et al., 2014; Toh et al., 2018). Therefore, a protein-mediated mechanism may represent a more likely mode of action for the exosomes.

MSCs exosomal proteomes have the potential to regulate various key biological processes that are involved in tissue repair and regeneration (Lai et al., 2012). MSCs-derived exosomes could reduce the area of myocardial infarction in a mouse model of mydcardial ischemia-reperfusion injury through a protein-mediated mechanism (Lai et al., 2013). MSCs-derived exosomes also could attenuate hyperoxia-induced lung injury through the secretion of VEGF and TSG-6 (Braun et al., 2018; Chaubey et al., 2018). More studies are needed in the future to focus on the role of MSCs derived exosomal proteome in ALI/ARDS.

Although there are a great diversity of different techniques to isolate, characterize and quantify EVs. EVs are heterogeneous in size and content, and lack specific markers to distinguish EV subtypes (Théry et al., 2018). Therefore, classification and isolation of exosomes remains a challenge. In addition, the source of MSCs used to separate EV is significantly different. MSCs-derived exosomes from different tissue sources may have different characteristics. Exosomes derived from human MSCs from adipose tissue exhibited particularly stronger effects in promoting macrophage M2 polarization, inhibiting proinflammatory cytokine production and secretion, attenuating lung histopathological changes, and improving survival of sepsis-induced ALI mice than that of human bone marrow MSCs-exosomes and human umbilical cord MSCs-exosomes (Deng et al., 2022). Finally, how to produce exosomes on a large scale is also an issue that needs to be addressed. New strategies and more studies are needed to adequately classify and separate EV subpopulations with high accuracy, and heterogeneity of MSCs-EVs needs to be addressed prior to its clinical introduction.

## Clinical trial for ARDS/ALI by MSC therapy

Recent studies have demonstrated that mesenchymal stromal cells (MSCs) modulate the immune response and reduce lung injury in humans. A prospective, double-blind, multicentre, randomised trial demonstrated that MSCs could significantly decrease the plasma concentrations of angiopoietin two and C-reactive proteins in ARDS patients compared with the control group (Matthay et al., 2019b). Mortality at 28 and 60 days was numerically but not statistically higher in the MSC group than in the placebo group (Matthay et al., 2019b). Bellingan et al. reported that MSCs reduced mortality and increased ventilator-free and ICU-free days compared to placebo (Bellingan et al., 2019). However, another study showed that administration of allogeneic AD-MSCs siginificantly improved oxygenation index in ARDS patients, but parameters such as ventilator-free and ICU-free days and serum IL-6 and IL-8 levels did not show a difference (Zheng et al., 2014). In addition, the coronavirus disease 2019 (COVID-19), caused by severe acute respiratory syndrome coronavirus 2 (SARS-CoV-2), has brought a severe global public burden on health authorities. Excessive inflammation and the cytokine storm are regarded as major causes of ARDS due to severe COVID-19 (Vabret et al., 2020). Since the outbreak of the COVID-19 pandemic, a number of MSCs-therapy clinical trials has been conducted. Transplanting MSCs in COVID-19 patients has immunomodulatory effects that can prevent the cytokine storm and decrease the tissue damage (Leng et al., 2020). Häberle H et al. reported that MSCs infusion is a safe treatment for COVID-19 ARDS that improves pulmonary function and overall outcome in this patient population (Häberle et al., 2021). Shi et al. performed a phase 2 trial to assess the efficacy and safety of MSCs to treat severe COVID-19 patients with lung damage, MSCs administration exerted numerical improvement in whole lung lesion volume and significantly reduced the proportions of solid component lesion volume compared with the placebo, the 6-min walk test showed an increased distance in the MSCs-treated group (Shi et al., 2021b). Lanzoni G et al. found that inflammatory cytokines were significantly decreased in UC-MSC-treated subjects at day 6 and patient survival was improved (Lanzoni et al., 2021). Another prospective long-term follow-up study showed that MSCs medication not only achieved a beneficial short-term effect but also exerted a long-term therapeutic benefit on lung lesions with good tolerance in severe COVID-19 patients (Shi et al., 2022). These data suggested that MSCs treatment was safe and may be beneficial to COVID-19 patients.

In addition, MSCs-derived exosomes could improve patients’ clinical status and oxygenation, reduce neutrophil count and lymphopenia, and decrease acute phase reactants such as C-reactive protein and ferritin (Sengupta et al., 2020). Therefore, MSCs-derived exosomes may be a promising therapy for COVID-19 patients due to its safety and capacity to downregulate cytokine storm.

Further optimization of the type of MSCs and their derived exosomes to be infused, the viability of MSCs before infusion, the dose, route and interval of administration of MSCs and their derived exosomes. Meanwhile, more multicenter, randomized, controlled trials and long-term follow-up studies are needed to validate the efficacy of MSCs and their derived exosomes in the treatment of COVID-19 patients. In addition, it remains to be developed with appropriate manufacturing and quality control regulations, and carefully evaluate the use of EVs through rational clinical trial design and appropriate supervision.

## Conclusion and perspectives

ALI/ARDS is a severe acute respiratory syndrome with high mortality. At present, there is no effective treatments for ARDS. Based on their tissue-repair, anti-inflammation, anti-apoptosis, and immunomodulation effects, MSCs have opened new avenues for ALI/ARDS treatment. However, the mechanisms of MSCs in the treatment of ALI/ARDS are complex and not fully understood. For example, MSCs can exert therapeutic effects through homing, multidirectional differentiation, paracrine signaling, and exosomes. In addition, the therapeutic effects of MSCs in ALI/ARDS differ based on the degree of lung injury, the time of lung injury, and various pathophysiological factors.

Currently, clinical evidence is insufficient to demonstrate the safety and effectiveness of MSCs, which have the advantages of low immunogenicity, strong *in vitro* amplification, stable genetic characteristics, and easy accessibility. However, MSCs also have drawbacks such as tumorigenicity and gene mutation after transplantation.

The cell survival and homing efficiency of MSCs are also key issues that should be the focus of future research, and many issues must be addressed before MSCs can be widely applied in clinical applications. For example, the mechanisms of MSCs require further basic and clinical studies, as do the interactions of stem cells with injured lung tissues.

In conclusion, regenerative medicine has broad prospects in the treatment of ALI/ARDS, although we must be vigilant in terms of safety during the treatment process.
